# Correction: Employment quality and mental health in China under the policy of expanding jobs and benefiting people's livelihood: gender differences between low-quality employment and work-family values

**DOI:** 10.3389/fpubh.2026.1863341

**Published:** 2026-04-28

**Authors:** Frontiers Production Office

**Affiliations:** Frontiers Media SA, Lausanne, Switzerland

**Keywords:** employment quality, standard employment, mental health, China, gender differences

Reviewer Rui Zhao was erroneously omitted as a reviewer. The reviewer's details are:

Rui Zhao, Xi'an Jiaotong University Health Science Center, China

The received date erroneously appeared as “16 September 2025”. It should be “01 August 2025”.

The original version of this article has been updated.

