# Supplementary material for: Employment quality and mental health in China under the policy of expanding jobs and benefiting people’s livelihood: gender differences between low-quality employment and work-family values
Source: Front Public Health. 2025 Nov 25;13:1677809. doi: 10.3389/fpubh.2025.1677809 (PMC12685863; doi:10.3389/fpubh.2025.1677809)

Appendix

Text A. Items MCS

The specific items for the MCS are:

For general mental health:

1. "During the past 4 weeks, how often did you feel down or gloomy?
2. "During the past 4 weeks, how often did you feel calm and relaxed?

For vitality

1. "During the past 4 weeks, how often did you feel energetic?

For role emotional

1. "During the past 4 weeks, how often did you feel that due to mental health or emotional problems – you achieved less than you wanted to at work or in everyday activities?
2. "During the past 4 weeks, how often did you feel that due to mental health or emotional problems – you carried out your work or everyday tasks less thoroughly than usual?

For social functioning:

1. "During the past 4 weeks, how often did you feel that due to physical or mental health problems you were limited socially, that is, in contact with friends, acquaintances, or relatives?

**Table A.** Mean MCS in 2016 and 2018 and mean change in MCS (range 0 to 100) stratified by gender (N= 7406 weighted)

|  | **Men** |  | **Women** |  | **Sig.** |
| --- | --- | --- | --- | --- | --- |
| **N** | 3501 |  | 3905 |  |  |
|  | **Mean** | **s.d.** | **Mean** | **s.d.** |  |
| MCS 2016 | 53.90 | 0.34 | 51.95 | 0.34 | <.002 |
| MCS 2018 | 52.74 | 0.35 | 51.33 | 0.36 | <.002 |

**Table B.** Sample characteristics and descriptive statistics (percentages) stratified by gender (N= 7406 weighted)

|  | **Men** |  | **Women** |  | **Sig.** |
| --- | --- | --- | --- | --- | --- |
|  | **3,501** |  | **3,905** |  |  |
| **EQ Clusters** | N | % | N | % |  |
| SER-like jobs | 1,490 | 42.6 | 809 | 20.7 | .000 |
| Precarious unsustainable jobs | 187 | 5.3 | 1,105 | 28.2 |  |
| Precarious full-time jobs | 582 | 1.66 | 449 | 11.4 |  |
| SER-light jobs | 697 | 1.99 | 551 | 14.1 |  |
| Portfolio jobs | 460 | 1.31 | 150 | 3.8 |  |
| Protected part-time jobs | 85 | 2.4 | 841 | 21.5 |  |
| **Important to be successful at work** |  |  |  |  |  |
| Not so/unimportant | 735 | 21 | 1015 | 26 | <.002 |
| Important | 2,100 | 60 | 2,343 | 60 |  |
| Very important | 666 | 19 | 547 | 14 |  |
| **Important to have children** |  |  |  |  |  |
| Not so/unimportant | 490 | 14 | 429 | 11 | <.002 |
| Important | 1,120 | 32 | 1015 | 26 |  |
| Very important | 1,891 | 54 | 2,461 | 63 |  |
| **Age** |  |  |  |  | .028 |
| Under 30 | 315 | 9 | 273 | 7 |  |
| 30-39 | 700 | 20 | 859 | 22 |  |
| 40-49 | 1,155 | 33 | 1,289 | 33 |  |
| 50-59 | 1,050 | 30 | 1,171 | 30 |  |
| 60+ | 281 | 8 | 313 | 8 |  |
| **Nationality** |  |  |  |  | <.002 |
| Not native | 420 | 12 | 351 | 9 |  |
| Native | 3,081 | 88 | 3,554 | 91 |  |
| **Region** |  |  |  |  | .077 |
| West Germany | 2,800 | 80 | 3046 | 78 |  |
| East Germany | 701 | 20 | 859 | 22 |  |
| **Occupational class (ISCO)** |  |  |  |  | <.002 |
| Upper service class | 700 | 20 | 390 | 10 |  |
| lower service class | 875 | 25 | 1,171 | 30 |  |
| routine non manual | 455 | 13 | 1,680 | 43 |  |
| skilled and unskilled manual | 1,505 | 43 | 664 | 17 |  |
| **Educational attainment** | | | |  | .516 |
| no vocational training | 245 | 7 | 234 | 6 |  |
| vocational training | 2,240 | 64 | 2,538 | 65 |  |
| Higher education | 1016 | 29 | 1,093 | 28 |  |
| **Household composition** |  |  |  |  | <.002 |
| single household | 350 | 10 | 430 | 11 |  |
| single parent | 70 | 2 | 273 | 7 |  |
| no kids, partner | 1,295 | 37 | 1,562 | 40 |  |
| Kids, partner | 1,786 | 51 | 1,640 | 42 |  |
| **Second job** |  |  |  |  | .266 |
| No | 3,256 | 93 | 3,592 | 92 |  |
| Yes | 245 | 7 | 313 | 8 |  |

**Table C.** Comparison of selected fit indices and degree of model improvement over the different latent class models, CFPS 2016

| **Model** | **BIC** | **AIC** | **CAIC** | **Change in BIC** | **Change in AIC** | **Change in CAIC** |
| --- | --- | --- | --- | --- | --- | --- |
| 1 cluster | 255327.9 | 255220.7 | 255339.9 |  |  |  |
| 2 clusters | 238942.5 | 238748 | 238966.5 | 17496.48 | 17593.7 | 17484.5 |
| 3 clusters | 235739.4 | 235447.8 | 235775.4 | 3314.158 | 3311.387 | 3321.2 |
| 4 clusters | 234146.2 | 233757.4 | 234164.9 | 1704.292 | 1701.521 | 1721.6 |
| 5 clusters | 232912.0 | 232426.9 | 232972.0 | 1344.359 | 1341.588 | 1303.0 |
| 6 clusters | 232437.5 | 231854.2 | 232509.5 | 486.2669 | 583.8458 | 474.6 |
| 7 clusters | 231978.9 | 231298.3 | 232097.2 | 469.7054 | 566.9243 | 423.4 |
| 8 clusters | 231847.0 | 231070.2 | 231788.7 | 142.1299 | 239.2487 | 299.6 |

**Table D.** Prevalence of work/family value and socio-demographic characteristics for each of the employment-quality type (column percentages and Bonferroni corrected p-values) stratified by gender (N=7,237 weighted)

|  | **Men** | | | | | | **Women** | | | | | |
| --- | --- | --- | --- | --- | --- | --- | --- | --- | --- | --- | --- | --- |
|  | **SE** | **PU** | **PF** | **SL** | **PO** | **PP** | **SE** | **PU** | **PF** | **SL** | **PO** | **PP** |
| **Work value orientation** |  |  |  |  |  |  |  |  |  |  |  |  |
| Not so/(un)important | 25 | 28 | 16† | 19† | 19 | 44† | 22 | 32† | 20 | 22 | 13 | 34† |
| Important | 62 | 55 | 62 | 62 | 59 | 48 | 65 | 59 | 59 | 63 | 64 | 60 |
| Very important | 17 | 19 | 24† | 23† | 26† | 11 | 16 | 12 | 25† | 18 | 26† | 9† |
| **Family value** | | | | | | |  |  |  |  |  |  |
| Not so/(un)important | 13 | 20 | 17 | 16 | 11 | 27† | 22 | 7† | 12† | 16 | 15 | 6† |
| Important | 32 | 39 | 37 | 32 | 31 | 37 | 23 | 26 | 34† | 32† | 28 | 26 |
| Very important | 58 | 43† | 49† | 55 | 61 | 39† | 58 | 70† | 58 | 56 | 60 | 72† |
| **Age** |  |  |  |  |  |  |  |  |  |  |  |  |
| Under 30 | 5 | 26† | 18† | 10† | 6 | 18† | 7 | 7 | 15† | 13† | 7 | 3† |
| 30-39 | 20 | 22 | 25 | 23 | 19 | 22 | 20 | 25 | 28† | 18 | 21 | 22 |
| 40-49 | 37 | 22† | 29† | 35 | 38 | 26 | 31 | 35 | 29 | 31 | 28 | 43† |
| 50-59 | 34 | 25 | 25† | 31 | 34 | 30 | 35 | 28† | 25† | 36 | 40 | 30 |
| 60+ | 10 | 10 | 9 | 7 | 8 | 9 | 12 | 10 | 8 | 8 | 9 | 7† |
| **Nationality** |  |  |  |  |  |  |  |  |  |  |  |  |
| Not German | 8 | 24† | 23† | 14† | 10 | 2 | 5 | 15† | 15† | 8 | 9 | 7 |
| German | 94 | 78† | 79† | 88† | 93 | 100 | 97 | 87† | 87† | 94 | 93 | 95 |
| **Region** |  |  |  |  |  |  |  |  |  |  |  |  |
| West Germany | 88 | 73† | 69† | 78† | 83 | 72† | 79 | 84† | 62† | 72 | 79 | 87† |
| East Germany | 14 | 29† | 33† | 24† | 19 | 30† | 23 | 18† | 40† | 30 | 23 | 15† |
| **ISCO** |  |  |  |  |  |  |  |  |  |  |  |  |
| Upper service class | 31 | 6† | 7† | 8† | 33 | 16 | 26 | 5† | 7† | 6† | 23 | 9† |
| Lower service class | 34 | 18† | 12† | 17† | 32 | 36 | 49 | 19† | 21† | 28† | 49 | 35† |
| Routine | 13 | 19 | 12 | 17 | 11 | 23 | 24 | 50† | 48† | 51† | 23 | 50† |
| (Un)skilled manual | 27 | 62† | 74† | 62† | 29 | 29 | 5 | 30† | 28† | 19† | 9 | 10† |
| **Educational attainment** | | | |  |  |  |  |  |  |  |  |  |
| No vocational training | 4 | 20† | 14† | 10† | 7 | 2 | 2 | 13† | 10† | 4† | 2 | 6† |
| Vocational training | 58 | 64 | 79† | 82† | 50 | 65 | 47 | 73† | 75† | 76† | 34 | 71† |
| Higher education | 42 | 19† | 11† | 11† | 47 | 36 | 54 | 17† | 18† | 23† | 67† | 26† |
| **Household composition** | | |  |  |  |  |  |  |  |  |  |  |
| Single household | 9 | 21† | 16† | 13 | 6 | 22† | 15 | 9† | 16 | 17 | 16 | 8† |
| Single parent | 2 | 10† | 3 | 4† | 1 | 7† | 7 | 8 | 10 | 7 | 4 | 8 |
| Partner | 38 | 34 | 39 | 40 | 36 | 32 | 50 | 34† | 44 | 54 | 53 | 28† |
| Children, partner | 55 | 39† | 46† | 48† | 61 | 44 | 31 | 54† | 35 | 26 | 31 | 60† |
| **Second job** |  |  |  |  |  |  |  |  |  |  |  |  |
| No | 91 | 88 | 93 | 94 | 90 | 82 | 92 | 90 | 94 | 92 | 89 | 89 |
| Yes | 9 | 12 | 7 | 6  6 | 10 | 18 | 8 | 10 | 6 | 8 | 11 | 11 |
| † p-value is < 0.05 for Bonferroni-corrected comparison within the cluster type, reference is SER-like jobs. Abbreviations; SE: SER-like jobs; PU: Precarious unsustainable jobs; PF: Precarious full-time jobs; SL: SER-light jobs; PO: Portfolio jobs; PP: protected part-time jobs.  category. | | | | | | | | | | | | |

**Table E.** Lagged regression models between EQ clusters and mental health two years later, women and men (N=7406, weighted, listwise deletion of missing values).

|  | Model 1^a,b^ | Model 2^a,b^ |
| --- | --- | --- |
|  | b (C.I.) sig. | b (C.I.) sig. |
| **constant** | 25.777(241787-27.377)*** | 27.807(26.145-29.469)*** |
| **EQ clusters** (SER-like, ref.) |  |  |
| Precarious unsustainable | -1.314 (-3.792-1.387)n.s. | -4.273 (-6.790--1.757)*** |
| Precarious full-time | -1.316 (-3.588-1.177)n.s. | 0.820 (-1.353-2.770)n.s. |
| SER-light | -0.799 (-2.990-1.624)n.s. | -2.265 (-4.306--0.224)* |
| Portfolio | 1.650 (-1.300-4.389)n.s. | -0.683 (-3.363-2.218)n.s. |
| Protected part-time | 3.305 (-0.575-6.964)(*) | 0.519 (-2.918-3.835)n.s. |
| **Importance of being succesful at work** |  |  |
| Not so/unimportant (ref.) | | |
| Important | 0.716 (-0.714-1.925)n.s. |  |
| Very important | 2.482 (0.813-4.152)** |  |
| **Importance of having children** | | |
| Not so/unimportant (ref.) | | |
| Important |  | -1.717 (-2.985--0.370)* |
| Very important |  | -1.943 (-3.354--0.733)** |
| **Interactions EQ typology*(family/work) value orientation** | | |
| Pre unsustainable*important | -1.755 (-4.832-1.544)n.s. | 3.665 (0.262-6.969)* |
| Pre unsustainable*very important | -0.964 (-4.923-3.318)n.s. | 2.228 (-1.319-5.552)n.s. |
| Precarious FT*important | 2.442 (-0.323-4.984)(*) | -0.997 (-3.47-1.679)n.s. |
| Precarious FT*very important | -0.456 (-3.702-2.912)n.s. | -0.997 (-3.47-1.679)n.s. |
| SER-light*important | -0.324 (-2.872-2.445)n.s. | 0.795 (-2.135-3.503)n.s. |
| SER-light*very important | -0.416 (-3.652-2.942)n.s. | 2.91 (0.453-5.550)* |
| Portfolio*important | 0.525 (-2.845-3.674)n.s. | 0.337 (-3.329-3.78)n.s. |
| Portfolio*very important | -3.819 (-7.479--0.159)* | 3.220 (-0.135-6.354)(*) |
| Protected PT*important | -5.939 (-10.592--1.485)* | -7.2 (-12.27--2.150)** |
| Protected PT*very important | -2.944 (-13.487-7.62)n.s. | 2.420 (-3.03-7.649)n.s. |
| **Gender (Men, ref.)** |  |  |
| women | 0.677(-1.469-2.60)n.s. | -1.265 (-2.865-0.557)n.s. |
| **Interactions EQ typology*gender** |  |  |
| Pre unsustainable*women | 0.532 (-2.825-3.667)n.s. | 4.138 (0.840-7.436)* |
| Precarious FT*women | -0.74 (-4.316-2.956)n.s. | -4.242 (-7.464--1.02)* |
| SER-light*women | 0.615 (-3.276-4.285)n.s. | 2.566 (-0.928-5.838)n.s. |
| Portfolio*women | -0.824 (-7.379-5.952)n.s. | -4.130 (-8.360-0.32)(*) |
| Protected PT*women | -1.572 (-5.827-2.906)n.s. | 2.846 (-1.627-6.996)n.s. |
| **Importance of work success*gender** | |  |
| important*women | -0.90 (-3.112-1.532)n.s. |  |
| very important*women | -1.090 (-4.164-2.205)n.s. |  |
| **Importance to have children*gender** | |  |
| important*women |  | 1.768 (-0.929-4.243)n.s. |
| very important*women |  | 1.409 (-0.895-3.490)n.s. |
| **3 way interaction: (family/work) value*EQ*gender** | |  |
| Precarious unsustainable*important*women | 2.387 (-1.656-6.207)n.s. | -4.109(-8.619-0.622)(*) |
| Precarious unsustainable*very important*women | -2.117(-7.309-3.297)n.s. | -2.887 (-6.964-1.512)n.s. |
| Precarious FT*important*women | -2.304 (-6.412-2.026)n.s. | 0.940(-3.663-5.32)n.s. |
| Precarious FT*very important*women | -2.575 (-7.788-2.859)n.s. | 3.077 (-1.170-7.103)n.s. |
| SER-light*important*women | 1.353 (-3.10-5.585)n.s. | -2.119 (-6.764-2.748)n.s. |
| SER-light*very important*women | 1.058(-4.818-6.713)n.s. | -1.44 (-5.550-2.889)n.s. |
| Portfolio*important*women | -3.133 (-10.25-4.206)n.s. | 3.280 (-3.189-9.526)n.s. |
| Portfolio*very important*women | 0.608 (-7.957-8.952)n.s. | 2.506 (-3.117-7.907)n.s. |
| Protected PT*important*women | 5.346 (0.123-10.569)* | 3.763 (-2.659-9.963)n.s. |
| Protected PT*very important*women | -2.339 (-13.527-9.07)n.s. | -4.533 (-10.579-1.753)n.s. |
| **MCS 2016** | 0.6 (0.580-0.632)*** | 0.616 (0.594-0.637)*** |
| (*) p ≤ 0,1; * p ≤ 0,05 ** p ≤ 0,01; *** p ≤ 0,001 | | |
| a Models are controlled for: age group nationality region education, occupational class, family composition and second job. | | |
| \| **Table F.** Linear Regression Results Predicting MCS at T2 (95% Confidence Interval) (N=231 men under 30) \| \| \| \| \| \| \| \| --- \| --- \| --- \| --- \| --- \| --- \| --- \| \|  \| **M1** \| **M2** \| **M3** \| **M4** \| **M5** \| **M6** \| \| Intercept \| 25.60*** \| 25.60*** \| 26.36*** \| 25.48*** \| 26.64** \| 26.37*** \| \| **EQ Clusters** \|  \|  \|  \|  \|  \|  \| \| *SER-like (ref.)* \|  \|  \|  \|  \|  \|  \| \| Precarious unsustainable \| -3 \| -3.16 \| -3.30 \| -3.07 \| -2.56 \| -7.72** \| \| Precarious full-time \| 0.42 \| -0.2 \| -0.16 \| 0.16 \| -15.20 \| -3.0 \| \| SER-light \| -1.2 \| -1.19 \| -1.17 \| -1.15 \| -4.95 \| -8.73* \| \| Portfolio \| -3.90 \| -5.06* \| -5.06* \| -4.92* \| -2.85 \| -19.09*** \| \| Protected part-time \| -9.43*** \| -9.58*** \| -9.63*** \| -9.55*** \| -3.42 \| -10.93** \| \| Mental health score in 2016 \| 0.64*** \| 0.64*** \| 0.64*** \| 0.64*** \| 0.65*** \| 0.68*** \| \| Importance of work success \| \|  \|  \|  \|  \|  \| \| *Not so/unimportant (ref.)* \| \|  \|  \|  \|  \|  \| \| Important \|  \|  \| -1.08 \|  \| -3.58 \|  \| \| Very important \|  \|  \| -1.17 \|  \| -1.96 \|  \| \| Importance to have children \| \|  \|  \|  \|  \|  \| \| *Not so/unimportant (ref.)* \| \|  \|  \|  \|  \|  \| \| Important \|  \|  \|  \| 0.20 \|  \| -8.93* \| \| Very important \|  \|  \|  \| 0.65 \|  \| -3.69 \| \| Interactions EQ clusters and values \| \|  \|  \|  \|  \|  \| \| Precarious unsustainable*Important \| \|  \|  \|  \| -5.06 \| 11.35* \| \| Precarious unsustainable*Very important \| \|  \|  \|  \| -1.80 \| 3.67 \| \| Precarious full-time*Important \| \|  \|  \|  \| 16.52 \| 8.44* \| \| Precarious full-time*Very important \| \|  \|  \|  \| 13.3 \| 3.39 \| \| SER-light*Important \|  \|  \|  \|  \| 3.34 \| 15.85** \| \| SER-light*Very important \| \|  \|  \|  \| -12.03 \| 7.23 \| \| Portfolio*Important \|  \|  \|  \|  \| 2.27 \| 26.30*** \| \| Portfolio*Very important \| \|  \|  \|  \| 1.88 \| 13.84* \| \| Protected part-time*Important \| \|  \|  \|  \| -7.54 \| -10.20 \| \| Protected part-time*Very important \| \|  \|  \|  \| 3.0 \| 12.46* \| \| R2 \| 0.375 \| 0.422 \| 0.423 \| 0.423 \| 0.494 \| 0.527 \| \| (*) p ≤ 0,1; * p ≤ 0,05 ** p ≤ 0,01; *** p ≤ 0,001 \| \| \|  \|  \|  \|  \|   Model 1 shows interactions between EQ clusters, gender, and “importance of work success”; Model 2 shows interactions between EQ clusters, gender, and “importance of having children” | | |

­­­­­­

| **Table G.** Linear Regression Results Predicting MCS at T2 (95% Confidence Interval) (N=263 women under 30) | | | | | | |
| --- | --- | --- | --- | --- | --- | --- |
|  | **M1** | **M2** | **M3** | **M4** | **M5** | **M6** |
| Intercept | 43.47*** | 45.15*** | 44.29*** | 44.90*** | 51.54*** | 43.69*** |
| EQ Clusters |  |  |  |  |  |  |
| *SER-like (ref.)* |  |  |  |  |  |  |
| Precarious unsustainable | -9.43*** | -5.40 | -5.36 | -4.52 | 2.19 | -2.74 |
| Precarious full-time | -7.23* | -3.42 | -3.42 | -3.33 | -2.2 | -13.60 |
| SER-light | -6.17 | -0.6 | -0.42 | -0.94 | -9.65 | -1.38 |
| Portfolio | -13.06** | -14.81*** | -14.01*** | -14.04** | -59.40 | -25.51*** |
| Protected part-time | -7.2 | -5.37 | -5.05 | -5.09 | -15.43* | -6.79 |
| Mental health score in 2016 | 0.34** | 0.39*** | 0.39*** | 0.39*** | 0.34** | 0.47*** |
| Importance of work success | |  |  |  |  |  |
| *Not so/unimportant (ref.)* | |  |  |  |  |  |
| Important |  |  | 0.90 |  | -2.26 |  |
| Very important |  |  | 0.22 |  | -12.90 |  |
| Importance to have children | |  |  |  |  |  |
| *Not so/unimportant (ref.)* | |  |  |  |  |  |
| Important |  |  |  | 2.96 |  | -9.24 |
| Very important |  |  |  | 0.38 |  | -3.52 |
| Interactions EQ clusters and values | |  |  |  |  |  |
| Precarious unsustainable*Important | |  |  |  | -10.57 | 8.4 |
| Precarious unsustainable*Very important | |  |  |  | 2.52 | -5.29 |
| Precarious full-time*Important | |  |  |  | -3.28 | 16.27 |
| Precarious full-time*Very important | |  |  |  | 9.17 | 12.89 |
| SER-light*Important | |  |  |  | 7.40 | 8.93 |
| SER-light*Very important | |  |  |  | 83.7 | 1.76 |
| Portfolio*Important | |  |  |  | 24.25* | 39.76*** |
| Portfolio*Very important | |  |  |  | 43.66 | 22.95 |
| Protected part-time*Important | |  |  |  | 19.71* | 15.18 |
| Protected part-time*Very important | |  |  |  | -9.34 | 0.14 |
| R2 | 0.0203 | 0.302 | 0.303 | 0.313 | 0.412 | 0.400 |
| (*) p ≤ 0,1; * p ≤ 0,05 ** p ≤ 0,01; *** p ≤ 0,001 | | |  |  |  |  |

| **Table H.** Linear Regression Results Predicting MCS at T2 (95% Confidence Interval) (N=1,932 men between, 30 and 49) | | | | | | |
| --- | --- | --- | --- | --- | --- | --- |
|  | **M1** | **M2** | **M3** | **M4** | **M5** | **M6** |
| Intercept | 24.55*** | 24.65*** | 23.75*** | 26.68*** | 24.56*** | 26.93*** |
| EQ Clusters |  |  |  |  |  |  |
| *SER-like (ref.)* |  |  |  |  |  |  |
| Precarious unsustainable | -3.22** | -2.90* | -2.78* | -2.93* | -5.30* | -6.61** |
| Precarious full-time | 1.35 | 1.19 | 2 | 1.32 | 1.02 | 3.13 |
| SER-light | 1.25 | 0.88 | 0.76 | 0.90 | -2.47 | -0.40 |
| Portfolio | 2.45** | 2.48** | 2.15* | 2.52** | 1.40 | 0.33 |
| Protected part-time | 1.72 | 2.52 | 2.73 | 2.74 | 0.67 | 12.48** |
| Mental health score in 2016 | 0.64*** | 0.64*** | 0.62*** | 0.63*** | 0.62*** | 0.63*** |
| Importance of work success | |  |  |  |  |  |
| *Not so/unimportant (ref.)* |  |  |  |  |  |  |
| Important |  |  | 2.12*** |  | 0.5 |  |
| Very important |  |  | 2.69*** |  | 3.12** |  |
| Importance to have children |  |  |  |  |  |  |
| *Not so/unimportant (ref.)* |  |  |  |  |  |  |
| Important |  |  |  | -2.27*** |  | -0.79 |
| Very important |  |  |  | -1.66** |  | -3.09** |
| Interactions EQ clusters and values | |  |  |  |  |  |
| Precarious unsustainable*Important | |  |  |  | 4.05 | 3.68 |
| Precarious unsustainable*Very important | |  |  |  | 0.83 | 5.46 |
| Precarious full-time*Important | |  |  |  | 1.03 | -4 |
| Precarious full-time*Very important | |  |  |  | -2.28 | -1.92 |
| SER-light*Important |  |  |  |  | 4.55* | -2.75 |
| SER-light*Very important | |  |  |  | 1.56 | 4.08* |
| Portfolio*Important |  |  |  |  | 1.70 | -1.95 |
| Portfolio*Very important | |  |  |  | -1.2 | 5.13* |
| Protected part-time*Important | |  |  |  | 4.28 | -15.47** |
| Protected part-time*Very important | |  |  |  | -6.72 | -7.24 |
| R2 | 0.362 | 0.374 | 0.384 | 0.38 | 0.389 | 0.395 |
| (*) p ≤ 0,1; * p ≤ 0,05 ** p ≤ 0,01; *** p ≤ 0,001 | | |  |  |  |  |

| **Table I.** Linear Regression Results Predicting MCS at T2 (95% Confidence Interval) (N=2,055 women between, 30 and 49) | | | | | | |
| --- | --- | --- | --- | --- | --- | --- |
|  | **M1** | **M2** | **M3** | **M4** | **M5** | **M6** |
| Intercept | 27.51*** | 26.04*** | 27.33*** | 27.47*** | 26.69*** | 27.58*** |
| EQ Clusters |  |  |  |  |  |  |
| *SER-like (ref.)* |  |  |  |  |  |  |
| Precarious unsustainable | 0.95 | 1.36 | 1.34 | 1.4 | 0.19 | 4.11** |
| Precarious full-time | -1.35 | -0.82 | -0.88 | -0.70 | 1.85 | 0.36 |
| SER-light | 0.62 | 0.73 | 0.73 | 0.70 | 2.04 | -0.99 |
| Portfolio | 1.29 | 1.84 | 1.82 | 1.95 | 7.57* | 3.06 |
| Protected part-time | 1.50* | 1.39 | 1.38 | 1.48 | 2.44 | 3.2 |
| Mental health score in 2016 | 0.57*** | 0.57*** | 0.57*** | 0.57*** | 0.58*** | 0.56*** |
| Importance of work success |  |  |  |  |  |  |
| *Not so/unimportant (ref.)* |  |  |  |  |  |  |
| Important |  |  | -0.44 |  | -0.2 |  |
| Very important |  |  | 0.19 |  | 2.3 |  |
| Importance to have children |  |  |  |  |  |  |
| *Not so/unimportant (ref.)* |  |  |  |  |  |  |
| Important |  |  |  | -2 |  | 2.29 |
| Very important |  |  |  | -0.20 |  | -0.70 |
| Interactions EQ clusters and values | |  |  |  |  |  |
| Precarious unsustainable*Important | |  |  |  | 2.28 | -5.92** |
| Precarious unsustainable*Very important | |  |  |  | 0.18 | -2.12 |
| Precarious full-time*Important | |  |  |  | -2.7 | -3.46 |
| Precarious full-time*Very important | |  |  |  | -6.64** | -0.6 |
| SER-light*Important |  |  |  |  | -1.77 | -2.63 |
| SER-light*Very important |  |  |  |  | -1.55 | 4.24 |
| Portfolio*Important |  |  |  |  | -6.43 | -7.25 |
| Portfolio*Very important |  |  |  |  | -8.30 | 1.36 |
| Protected part-time*Important | |  |  |  | -0.80 | -5.31* |
| Protected part-time*Very important | |  |  |  | -4.86 | -0.79 |
| R2 | 0.326 | 0.338 | 0.338 | 0.339 | 0.346 | 0.347 |
| (*) p ≤ 0,1; * p ≤ 0,05 ** p ≤ 0,01; *** p ≤ 0,001 | | |  |  |  |  |

| **Table J.** Linear Regression Results Predicting MCS at T2 (95% Confidence Interval) (N=1,433men over 49) | | | | | | |
| --- | --- | --- | --- | --- | --- | --- |
|  | **M1** | **M2** | **M3** | **M4** | **M5** | **M6** |
| Intercept | 25.02*** | 26.94*** | 27.36*** | 28.14*** | 26.04*** | 27.67*** |
| EQ Clusters |  |  |  |  |  |  |
| SER-like (ref.) |  |  |  |  |  |  |
| Precarious unsustainable | -3.06** | -3.42** | -3.47** | -3.57** | -1.73 | -4.60* |
| Precarious full-time | -1.81* | -2.27* | -2.23* | -2.47** | -3.17 | -2.59 |
| SER-light | -3.48*** | -3.54*** | -3.52*** | -3.89*** | -1.65 | -4.42** |
| Portfolio | 0.73 | 0.8 | 0.76 | 0.59 | 0.99 | 4.0 |
| Protected part-time | 1.89 | 2.02 | 1.8 | 1.37 | 4.96 | 2.05 |
| Mental health score in 2016 | 0.63*** | 0.64*** | 0.64*** | 0.64*** | 0.63*** | 0.64*** |
| Importance of work success |  |  |  |  |  |  |
| Not so/unimportant (ref.) |  |  |  |  |  |  |
| Important |  |  | -0.79 |  | 0.24 |  |
| Very important |  |  | -0.42 |  | 0.88 |  |
| Importance to have children |  |  |  |  |  |  |
| Not so/unimportant (ref.) |  |  |  |  |  |  |
| Important |  |  |  | -1.88** |  | -1.7 |
| Very important |  |  |  | -1.08 |  | -0.67 |
| Interactions EQ clusters and values | |  |  |  |  |  |
| Precarious unsustainable*Important | |  |  |  | -2.35 | 3.15 |
| Precarious unsustainable*Very important | |  |  |  | -10.40 | 0.42 |
| Precarious full-time*Important | |  |  |  | 1.52 | 1.07 |
| Precarious full-time*Very important | |  |  |  | -2.79 | -0.99 |
| SER-light*Important |  |  |  |  | -3.60 | 0.75 |
| SER-light*Very important |  |  |  |  | 0.47 | 0.93 |
| Portfolio*Important |  |  |  |  | 1.04 | -5.18 |
| Portfolio*Very important |  |  |  |  | -2.54 | -3.8 |
| Protected part-time*Important | |  |  |  | -7.34 | 0.46 |
| Protected part-time*Very important | |  |  |  | 10. | -3.16 |
| R2 | 0.393 | 0.416 | 0.418 | 0.422 | 0.430 | 0.426 |
| (*) p ≤ 0,1; * p ≤ 0,05 ** p ≤ 0,01; *** p ≤ 0,001 | | |  |  |  |  |

| **Table K.** Linear Regression Results Predicting MCS at T2 (95% Confidence Interval) (N=1,521 women over 49) | | | | | | |
| --- | --- | --- | --- | --- | --- | --- |
|  | **M1** | **M2** | **M3** | **M4** | **M5** | **M6** |
| Intercept | 23.04*** | 25.83*** | 25.77*** | 25.84*** | 25.07*** | 25.67*** |
| EQ Clusters |  |  |  |  |  |  |
| *SER-like (ref.)* |  |  |  |  |  |  |
| Precarious unsustainable | -1.33 | -1.38 | -1.40 | -1.38 | -1.33 | -2.49 |
| Precarious full-time | -2.78* | -2.82* | -2.73* | -2.83* | -10.35*** | -4.33 |
| SER-light | 0.86 | 0.80 | 0.77 | 0.79 | 0.54 | 2.56 |
| Portfolio | -1.2 | -0.70 | -0.66 | -0.7 | -6.54 | -1.0 |
| Protected part-time | 1.27 | 1.08 | 1.06 | 1.09 | 2.36 | 2.19 |
| Mental health score in 2016 | 0.66*** | 0.65*** | 0.66*** | 0.65*** | 0.65*** | 0.66*** |
| Importance of work success |  |  |  |  |  |  |
| *Not so/unimportant (ref.)* |  |  |  |  |  |  |
| Important |  |  | -0.12 |  | -0.8 |  |
| Very important |  |  | -0.80 |  | 1.7 |  |
| Importance to have children |  |  |  | 0.2 |  | 0.32 |
| *Not so/unimportant (ref.)* |  |  |  | -0.16 |  | 0.19 |
| Important |  |  |  |  |  |  |
| Very important |  |  |  |  |  |  |
| Interactions EQ clusters and values |  |  |  |  |  |  |
| Precarious unsustainable*Important | |  |  |  | 0.30 | 2.2 |
| Precarious unsustainable*Very important | |  |  |  | -3.58 | 0.85 |
| Precarious full-time*Important |  |  |  |  | 9.87** | 0.69 |
| Precarious full-time*Very important | |  |  |  | 4.35 | 3.02 |
| SER-light*Important |  |  |  |  | 0.58 | -3.47 |
| SER-light*Very important |  |  |  |  | -1.26 | -1.56 |
| Portfolio*Important |  |  |  |  | 7.53 | 1.95 |
| Portfolio*Very important |  |  |  |  | 0.66 | -0.32 |
| Protected part-time*Important |  |  |  |  | -1.99 | -1.1 |
| Protected part-time*Very important | |  |  |  | -5.62 | -1.38 |
| R2 | 0.4 | 0.307 | 0.308 | 0.307 | 0.418 | 0.4 |
| (*) p ≤ 0,1; * p ≤ 0,05 ** p ≤ 0,01; *** p ≤ 0,001 | | |  |  |  |  |


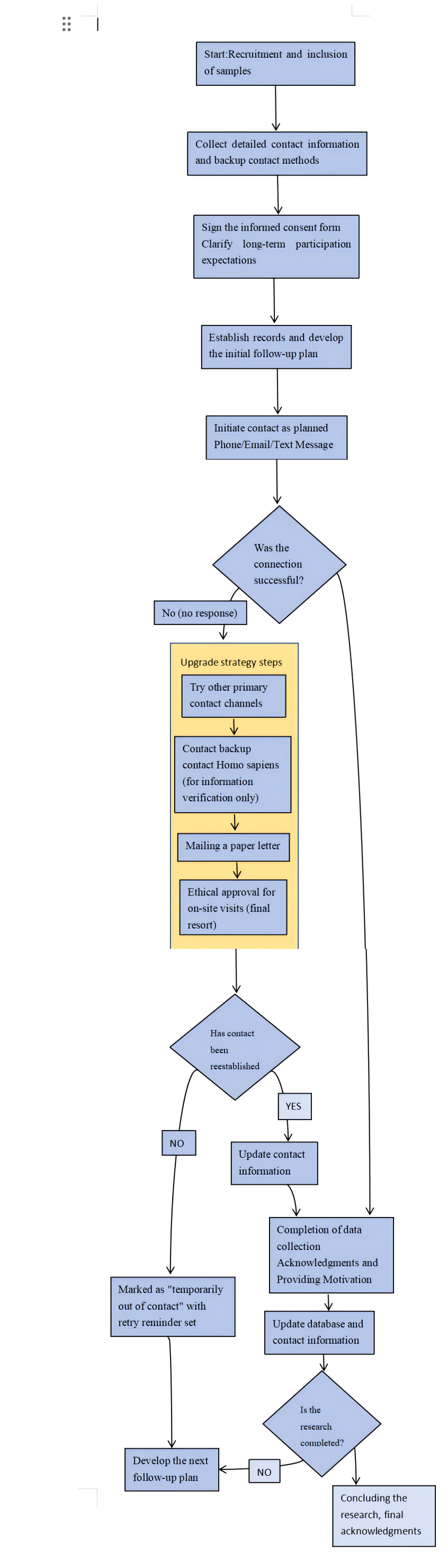

Supplement: Supplementary file 1 [file Data_Sheet_1.docx]
